# Spin glass behavior and magnetic boson peak in a structural glass of a magnetic ionic liquid

**DOI:** 10.1038/s41598-021-91619-z

**Published:** 2021-06-08

**Authors:** Maiko Kofu, Ryuta Watanuki, Toshiro Sakakibara, Seiko Ohira-Kawamura, Kenji Nakajima, Masato Matsuura, Takeshi Ueki, Kazuhiro Akutsu, Osamu Yamamuro

**Affiliations:** 1 J-PARC Center, Japan Atomic Energy Agency, Tokai, Ibaraki 319-1195 Japan; 2 Division of Materials Science and Chemical Engineering, Faculty of Engineering, Yokohama National University, Yokohama, Kanagawa 240-8501 Japan; 3 Institute for Solid State Physics, University of Tokyo, Kashiwa, Chiba 277-8581 Japan; 4 Comprehensive Research Organization for Science and Society, Tokai, Ibaraki 319-1106 Japan; 5 National Institute for Materials Science, Tsukuba, Ibaraki 305-0044 Japan; 6 Graduate School of Life Science, Hokkaido University, Sapporo, Hokkaido 060-0810 Japan

**Keywords:** Magnetic properties and materials, Magnetic properties and materials, Chemical physics

## Abstract

Glassy magnetic behavior has been observed in a wide range of crystalline magnetic materials called spin glass. Here, we report spin glass behavior in a structural glass of a magnetic ionic liquid, C4mimFeCl_4_. Magnetization measurements demonstrate that an antiferromagnetic ordering occurs at *T*_N_ = 2.3 K in the crystalline state, while a spin glass transition occurs at *T*_SG_ = 0.4 K in the structural glass state. In addition, localized magnetic excitations were found in the spin glass state by inelastic neutron scattering, in contrast to spin-wave excitations in the ordered phase of the crystalline sample. The localized excitation was scaled by the Bose population factor below *T*_SG_ and gradually disappeared above *T*_SG_. This feature is highly reminiscent of boson peaks commonly observed in structural glasses. We suggest the “magnetic” boson peak to be one of the inherent dynamics of a spin glass state.

## Introduction

Disordered magnetic materials often display spin freezing at finite temperature and are referred to as spin glasses (SGs)^1–3^. Theoretical attempts to model these systems have been made since the 1970s. It is generally accepted that randomness, frustration, and competing interaction are essential ingredients for SGs. These ingredients give rise to a complex energy landscape, many metastable valleys, or low-lying states in a phase space. Further, over-barrier hopping, which is time-, temperature- and magnetic-field-dependent, can occur between these valleys. In this respect, intrinsic slow relaxation takes place with a wide distribution of relaxation times. These include aging, memory, and rejuvenation effects, which have been studied extensively. However, the excitation characteristics to the metastable states are little understood. Is there an intrinsic excitation of the SG state?

Thus far, SG-like behavior has been reported in a variety of magnetic materials, including dilute magnetic alloys, mixed-phase magnetic oxides, intermetallics, and frustrated magnets, among others. Most target materials were crystalline, and SG states often appeared in proximity to long-range magnetic order phases. As the spin dynamics of crystalline materials are investigated, intrinsic spin dynamics that stemmed from the short-range magnetic order could remain even in the SG regime. To explore excitations inherent to the SG state experimentally, non-crystalline materials should be suitable.

A typical example of amorphous SGs is given in metallic glasses, for instance, multi-component alloys^4,5^ and binary intermetallic compounds synthesized by mechanical milling^6^. Some multi-component alloys are reentrant SGs in which a ferromagnetic ordered phase exists above the SG phase. In binary compounds, the possibility of microseparation is not excluded. Thus, short-range magnetic order can appear in these SG phases. In this article, we report a new type of SG material, a magnetic ionic liquid (MIL), C4mimFeCl_4_ (1-butyl-3-methylimidazolium tetrachloroferrate), that is nonmetallic, contains only one kind of magnetic element, and is completely vitrified without mechanical treatment.

**Figure 1:**
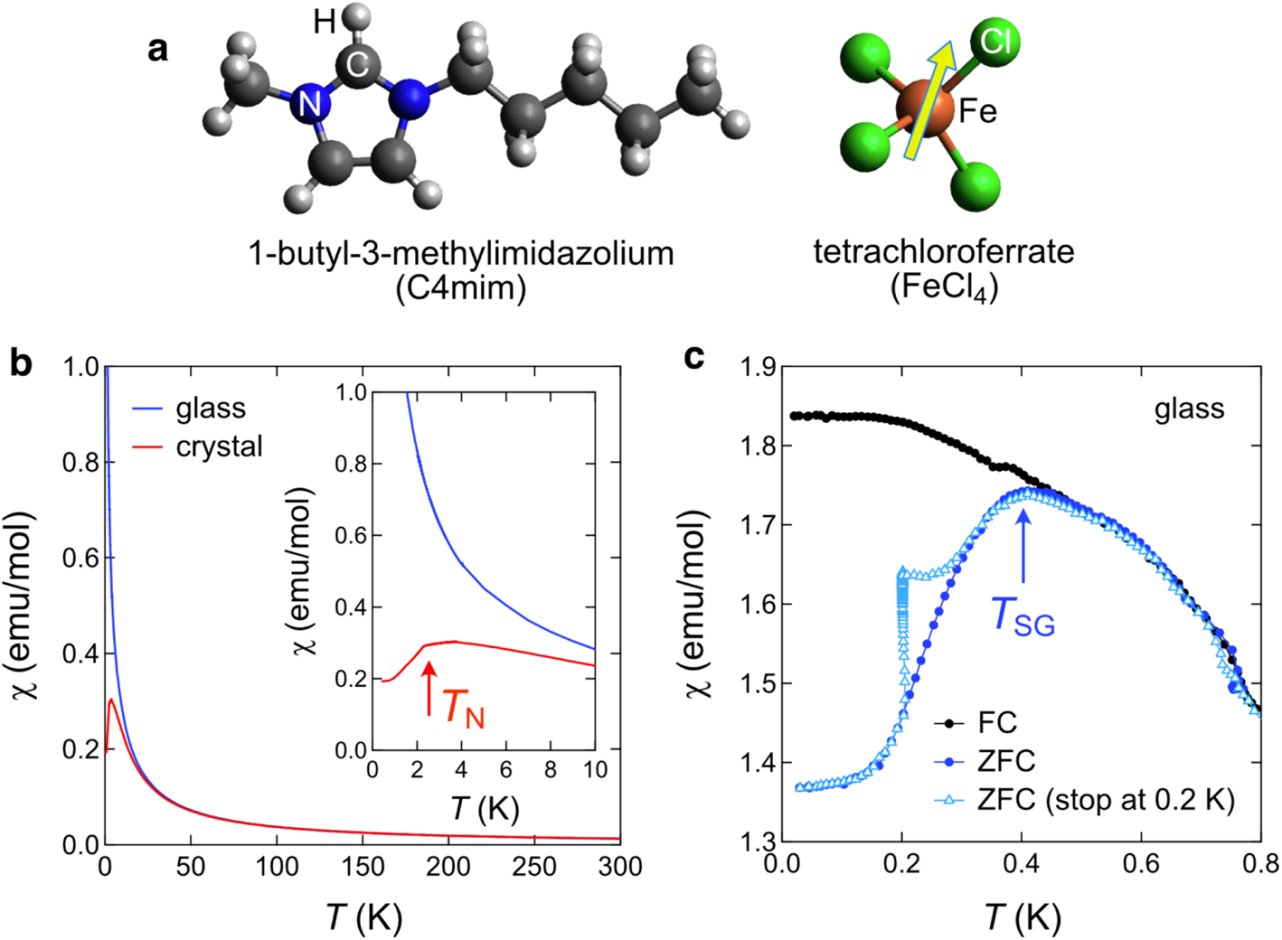
Magnetic properties of a magnetic ionic liquid (MIL). (**a**) Molecular structure of the MIL C4mimFeCl_4_, where the FeCl_4_^−^ anion has a spin of 5/2. (**b**) Temperature dependence of magnetic susceptibility in glassy and crystalline C4mimFeCl_4_. Inset shows an enlarged plot below 10 K. (**c**) Zero-field-cooled (ZFC) and field-cooled (FC) susceptibilities in the glassy state on heating at *T* ≤ 0.8 K. Open triangles represent magnetization with an intermittent stop for 50 min at *T* = 0.2 K. The glassy sample displays a spin glass transition at *T*_SG_ = 0.4 K, while the crystalline one exhibits an antiferromagnetic ordering at *T*_N_ = 2.3 K. All the susceptibility data were obtained under an applied magnetic field of 100 Oe.

MILs are a class of ionic liquids that exist in liquid state around room temperature and are one of the most attractive subjects in current materials science^7^. The paramagnetic behavior of C4mimFeCl_4_ was first demonstrated by Hayashi et al. in 2004^8^. C4mimFeCl_4_ comprises a large organic cation and a magnetic anion (Fig. 1a). After the discovery of the magnetic responsiveness of C4mimFeCl_4_, MILs have gained considerable attention owing to their versatile applications^9^. MILs solely contain ions, and hence are entirely distinct from conventional magnetic fluids, which are colloidal liquids made of ferromagnetic nanoparticles suspended in nonmagnetic fluids. Most researches focus on their liquid properties and studies on low-temperature magnetic properties are quite limited. One remarkable feature of C4mimFeCl_4_ is that either a crystalline or glassy state can be created depending on the thermal history. The glass transition and melting temperatures of C4mimFeCl_4_ are 182 K and 265 K, respectively^10^. We have investigated the magnetic properties of C4mimFeCl_4_ in both the structural glass and crystal states by magnetization and neutron scattering measurements.

Figure 1b presents magnetic susceptibilities as a function of temperature in both glassy and crystalline C4mimFeCl_4_. They display paramagnetic behavior above 20 K and are described by the Curie–Weiss law. The Weiss temperatures and effective paramagnetic moments were estimated to be − 3.58 K and 5.567 μ_B_ for the glassy state and − 4.08 K and 5.564 μ_B_ for the crystal state (Supplementary SI-3), respectively, which are in good agreement with previous reports^8,11^. The obtained effective moments are slightly smaller than the theoretical value for an isotropic Fe^3+^ ion in the high-spin state (*S* = 5/2), 5.9 μ_B_. The magnetism of C4mimFeCl_4_ is dominantly governed by the antiferromagnetic (AFM) interaction between the Fe^3+^ spins.

In the crystal state of C4mimFeCl_4_, an AFM transition occurs at *T*_N_ = 2.3 K (see the inset of Fig. 1b). Meanwhile, no anomaly was observed down to 2 K in the structural glass state. We made further measurements down to 0.03 K and found that the structural glass of C4mimFeCl_4_ exhibits an SG behavior (Fig. 1c). The zero-field-cooled (ZFC) magnetization shows a maximum at *T* ~ 0.4 K which we define as *T*_SG_, while the field-cooled (FC) magnetization decreases monotonously upon heating through *T*_SG_. Thus, there is a significant difference between the ZFC and FC magnetizations below *T*_SG_, which is usually observed around the SG transition. Furthermore, we investigated the waiting-time effect, which is a unique characteristic of the SG state^12–15^. After the ZFC process, the sample was heated to 0.2 K (< *T*_SG_) and remained at this temperature for *t*_w_ = 50 min. As *t*_w_ progressed, the magnetization increased, suggesting that the system relaxes to more stable states with lower energies. When heating is resumed, the magnetization increases again, but more gradually than the ZFC magnetization without the intermittent stops (open triangles in Fig. 1c). This behavior is referred to as the aging effect characterizing a spin freezing phenomenon. The time evolution of the magnetization in the aging regime was also investigated and is shown in Supplementary SI-4. The non-Debye relaxation process was demonstrated, suggesting a broad distribution of relaxation times.

**Figure 2:**
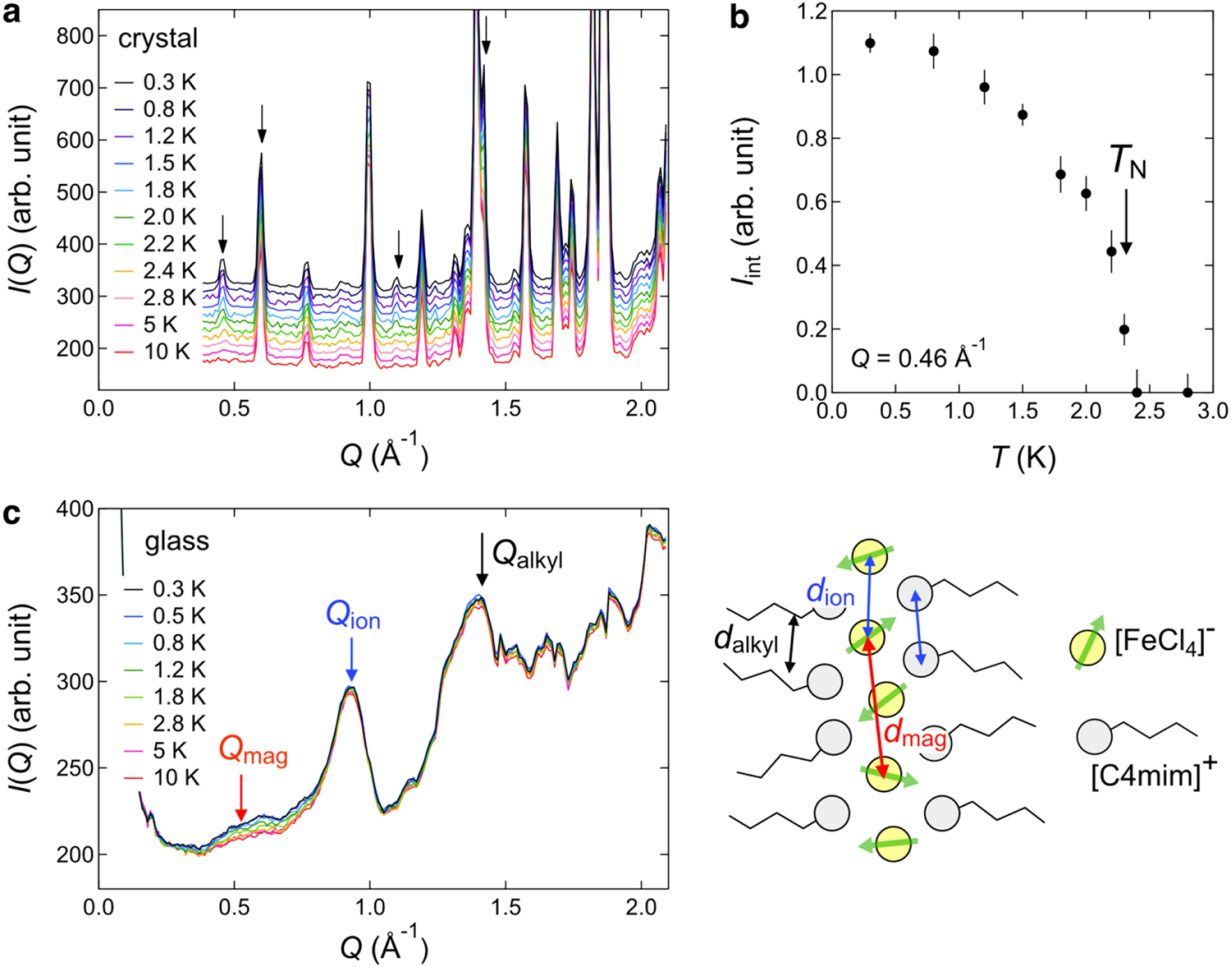
Diffraction patterns of C4mimFeCl_4_. (**a**) Diffraction patterns of crystalline C4mimFeCl_4_ between 0.3 and 10 K. Data are shifted upward for clarity. Arrows indicate the magnetic Bragg peaks that appeared below *T*_N_. (**b**) Temperature dependence of integrated intensity of the magnetic Bragg peak at *Q* = 0.46 Å^−1^. (**c**) Diffraction patterns of the structural glass of C4mimFeCl_4_ and schematic of the molecular and magnetic correlations with specific distances (*d* = 2π/*Q*).

To gain a deeper insight into the magnetic behavior within C4mimFeCl_4_, we performed neutron scattering experiments, which can provide energy (ℏω)- and momentum (*Q*)-resolved information. Here, deuterated C4mimFeCl_4_ was used to detect magnetic scattering without disturbance of strong incoherent scattering from H atoms (Supplementary SI-5). Figure 2 shows the *Q*-dependence of the scattering intensity at ℏω = 0, namely, the diffraction patterns. In the crystalline sample, additional magnetic Bragg peaks, indicated by the arrows in Fig. 2a, were observed at low temperatures. It is evident from Fig. 2b that the magnetic peaks appear below *T*_N_ = 2.3 K, which is compatible with the magnetic susceptibility data. On the other hand, the diffraction patterns of the glassy sample constitute broad peaks (Fig. 2c). Those at 0.9 Å^−1^ (= *Q*_ion_) and 1.4 Å^−1^ (= *Q*_alkyl_) have commonly been observed in imidazolium-based ionic liquids^16^, which have local lamellar structures comprising ionic layers (anions and the charged core parts of cations) and nonionic alkyl chain layers. Moreover, the above peaks are attributed to correlations between the anions (or charged core parts) in the ionic layers and between the alkyl chains in the nonionic layers, respectively (see the illustration in Fig. 2c). In addition to these structural peaks, temperature-dependent signals were found in the *Q* range of 0.3–0.9 Å^−1^. This diffuse signal increased upon cooling, suggesting the development of magnetic correlations. The center position of the diffuse peak (= *Q*_mag_) is approximately half of the *Q* value of the structural peak (*Q*_ion_ = 0.93 Å^−1^), indicating the AFM correlation between Fe^3+^ spins in FeCl_4_^−^ anions. It is also noted that the magnetic diffuse peak in the structural glass appears near the magnetic Bragg peaks in the crystalline sample, suggesting a similar local spin configuration in both the SG state and AFM phase (Supplementary SI-6).

**Figure 3:**
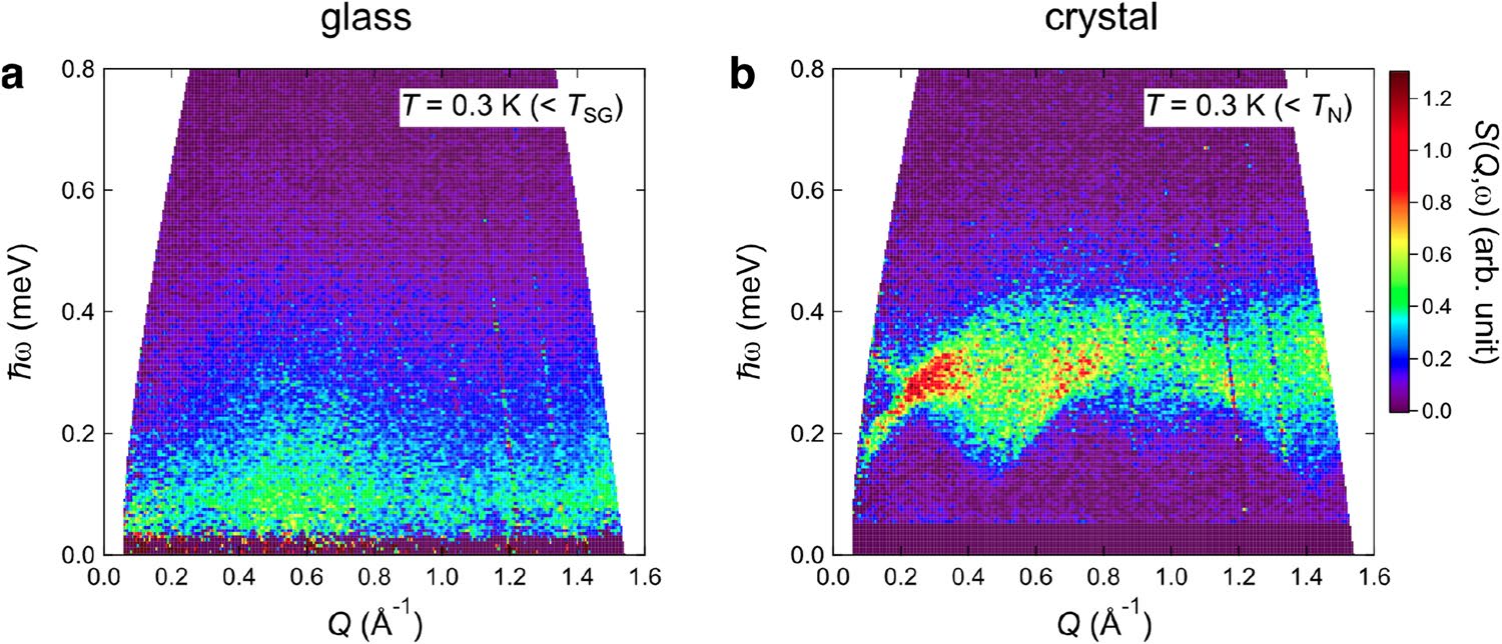
Magnetic excitations of C4mimFeCl_4_. (**a**,**b**) Energy (ℏω)-momentum (*Q*) maps of scattering intensities of (**a**) glass and (**b**) crystal states at *T* = 0.3 K.

We next describe the dynamical properties of C4mimFeCl_4_. Color contour maps of the dynamical structure factor *S*(*Q*,ω) are shown in Fig. 3. A distinct difference in the excitation spectra can be recognized between the glassy and crystal states. The glassy C4mimFeCl_4_ exhibits a broad low-energy magnetic excitation at 0.3 K (< *T*_SG_) (Fig. 3a). The scattering signal is enhanced at *Q* ~ 0.5 Å^−1^ and ℏω ~ 0.1 meV. Above *T*_SG_, the magnetic scattering is still clearly visible and its intensity is stronger near ℏω = 0 meV rather than near 0.1 meV (Supplementary SI-7), which is suggestive of the emergence of the magnetic relaxation process (quasielastic scattering). More specifically, the system exhibits a liquid-like behavior. In contrast, spin-wave excitations with a gap attributed to magnetic anisotropy were clearly observed in the AFM order phase of the crystalline sample (Fig. 3b). The excitations extended up to 0.5 meV, suggesting that the magnitude of magnetic interaction between the Fe^3+^ spins is of the order of 0.1 meV. The excitation spectrum drastically changes above *T*_N_ (Fig. S8); magnetic scattering extends over a wide ℏω-*Q* region. In other words, the Fe^3+^ spins fluctuate rapidly and the spin–spin correlations become weak, which is typical behavior for a magnetic order–disorder transition.

**Figure 4:**
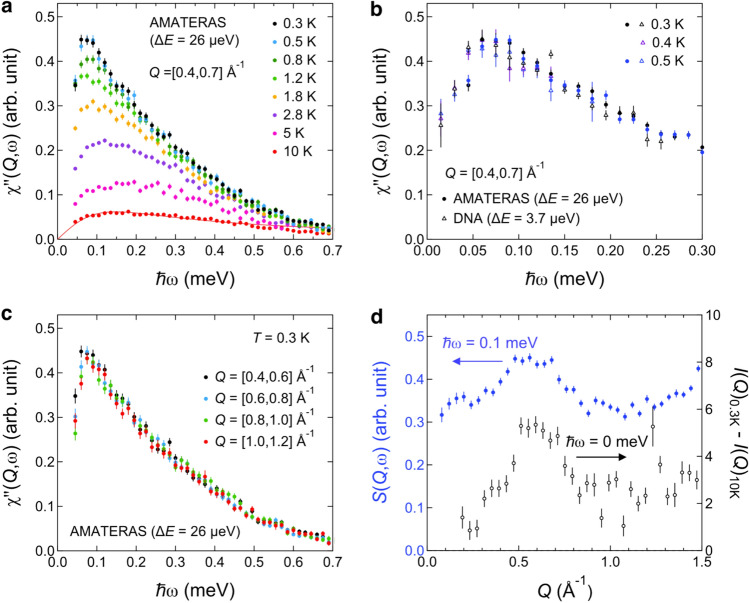
Energy, momentum and temperature dependence of magnetic excitation of glassy C4mimFeCl_4_. (**a**) Energy spectra corrected for the Bose factor, χ′′(*Q*,ω), in the glassy state in the temperature range of 0.3–10 K. Red solid curve represents the result of fit with the Debye relaxation model (see main text). (**b**) Energy spectra below *T* ≈ *T*_SG_ obtained using two spectrometers with different energy resolutions (∆*E*). (**c**) Energy spectra at *T* = 0.3 K with different *Q*, where scale factors are multiplied for comparison. (**d**) Constant-ℏω cuts at 0.1 ± 0.04 meV (excitation peak) and 0 ± 0.015 meV (elastic scattering) for the 0.3 K data, where the magnetic elastic scattering was obtained by subtracting the data at 10 K.

The spin dynamics of the SG state is summarized in Fig. 4. The raw energy spectra, results of the crystal state, and contribution of nuclear scattering are discussed in Supplementary SI-8 and SI-9. A striking result was found in the temperature evolution of the spectra corrected for the Bose thermal population factor, χ′′(*Q*,ω) = *S*(*Q*,ω)/(1 +  <*n*>) with < *n* >  = [exp(ℏω/*k*_B_*T*) − 1]^−1^. The spectrum at 0.3 K exhibits a peak at ~ 0.1 meV with a high energy tail and remains almost the same at 0.5 K (Fig. 4a), although a difference was observed in the uncorrected *S*(*Q*,ω) between 0.3 K and 0.5 K (Fig. S9). These data are taken in the measurement condition with an energy resolution of 26 μeV. To confirm this experimental observation, the spectra down to 0.02 meV were investigated using another spectrometer with a fine resolution of 3.7 μeV. Figure 4b shows the energy spectra below *T*_SG_ obtained with the two spectrometers. As a result, the excitation spectrum has a maximum at 0.06 meV and is scaled by the Bose factor below *T* ≈ *T*_SG_.

As the temperature increases above *T*_SG_, the spectrum significantly changes (Fig. 4a). The magnitude of χ”(*Q*,ω) decreases and the peak top position shifts toward the higher energy side. This result indicates that some sort of magnetic relaxation process is activated above *T*_SG_ and accelerates with increasing temperature. The spectrum at 10 K was fitted with a Debye relaxation model (the red solid curve in Fig. 4a), χ′′(ω) = χ′Γℏω/[(ℏω)^2^ + Γ^2^], where χ′ is the static susceptibility and Γ is the relaxation rate. The value of Γ derived from the fit is 0.149(4) meV, which corresponds to the relaxation time of 4.43(12) ps. The Debye relaxation does not completely reproduce the actual spectrum at 10 K, suggesting a distribution of relaxation times. Details of the magnetic relaxation processes in C4mimFeCl_4_, such as its temperature evolution and deviation from Debye behavior, will be presented in a future paper.

The energy spectrum at several *Q* positions is shown in Fig. 4c, where suitable scale factors are multiplied to compare the spectral shapes. Apparently, the spectral shape is independent of *Q*, suggesting that the magnetic excitation is a nondispersive localized mode. Given that spin clusters with short-range AFM correlations are formed in the SG state, magnons are enclosed in the clusters, giving rise to localized magnetic excitations. The excitation intensity at ℏω = 0.1 meV is enhanced at *Q* ~ 0.6 Å^−1^, at which a magnetic diffuse peak appears in the elastic channel (Fig. 4d). The magnetic scattering at ℏω = 0 and 0.1 meV stem from common spin correlations in the SG state.

We mention here that the observed feature is distinct from magnetic excitations in nanomagnets exhibiting a superparamagnetic behavior. Sharp and well-defined excitation peaks were observed in the nanomagnets, such as Mn_12_-acetate^17^, having a single type of spin cluster. The temperature dependence of the excitation intensity is described by the Boltzmann distribution, in contrast to C4mimFeCl_4_ whose intensity is scaled by the Bose factor; upon heating, the scattering intensity in ℏω > 0 decreases in the nanomagnets, while increases in C4mimFeCl_4_ (see Fig. S9). The slow relaxation of nanomagnets is caused by double-well potential and hence there are two stable states. On the other hand, the dynamics of spin glass is characterized by the multi-valley energy landscape. The Bose-scaled broad excitation observed in C4mimFeCl_4_ is suggestive of the complex energy landscape with a multitude of metastable states.

The localized magnetic excitation observed below *T*_SG_ is highly reminiscent of the “boson peak”^18^ universally observed in structural glasses below the glass transition temperatures in neutron and Raman spectroscopies. The peak energy is located at a few meV and does not vary with *Q*. Additionally, the excitation intensity is enhanced at the structural peak positions, and is scaled by the Bose factor, indicating the excitations are of harmonic oscillators. The microscopic origin of the boson peak is, however, still under debate. The low-energy magnetic excitation in the SG state of C4mimFeCl_4_ is similar to the structural boson peak and could be termed the “magnetic boson peak”. Incidentally, the structural boson peak is also seen in C4mimFeCl_4_. An excitation peak at 2.6 meV was found at higher *Q* and higher temperatures than the magnetic boson peak (Supplementary SI-10). The energy of the structural boson peak is roughly 40 times larger than that of the magnetic one because the molecular interaction is much stronger than the magnetic one in this system.

It is important to make a comparison with other SG systems studied by neutron scattering. A dilute magnetic alloy Cu_1−*x*_Mn_*x*_, known as a prototypical classical SG system, has been intensively investigated^19–22^. Magnetic scattering appears at *Q* positions corresponding to ferromagnetic clustering and spin-density-wave above and below *T*_SG_. The scattering around the magnetic diffuse peak positions is centered at ℏω = 0 and extends to energies of ~ 10 meV. The observed spectra were analyzed as a quasielastic scattering and explained by a model of dynamically fluctuating spin clusters with a broad distribution of relaxation times. No evidence for magnetic excitations has been adduced.

Spin dynamics of some metallic alloys exhibiting a reentrant SG behavior have also been studied^23–26^. Spin waves and quasielastic scattering are observed in the FM phase above *T*_SG_. Below *T*_SG_, the central peak at ℏω = 0, or a resolution-limited quasielastic peak, is dominant and spin-wave-like excitations sometimes appear due to ferromagnetically correlated magnetic clusters. Examining modern SG materials, including high-*T*c cuprates^27,28^, heavy fermions^29^, and frustrated magnets^30^, spin excitations are often observed in their SG states. Although the excitations are modified in the SG states, they exist even outside the SG phase. Therefore, excitations inherent in SG would be invisible or absent in SG states close to magnetic order phases.

To our knowledge, a Bose-scaled localized excitation was reported only in a magnetic quasicrystal Zn-Mg-Tb^31^. The energy spectrum and complicated *Q*-structure are well-described by a dodecahedral spin cluster model. The result is similar to that of the spin dynamics in structurally glassy C4mimFeCl_4_. It, however, should be noted that the spectrum of C4mimFeCl_4_ displays a much broader tail in the high energy side (Fig. 3a). This could be attributed to a number of spin clusters with different sizes and spin configurations.

Considering theoretical calculations, many attempts have been made to derive a spin-wave spectrum, predominantly for metallic systems, in the early stage of SG research^1,3^. Most calculations, however, have failed to detect spin waves in the SG regime except some specific models such as a planar SG^32,33^. Meanwhile, a computer simulation for dilute Ruderman-Kittel-Kasuya-Yosida-coupled spin systems with Heisenberg spins (e.g., Cu_1−*x*_Mn_*x*_) has shown that the excitation spectrum extends continuously from zero to the interaction energy of nearest-neighbor pairs and exhibits a peak in a low energy range. The spectrum can reproduce the specific heat of Cu_1-*x*_Mn_*x*_ (*x* ~ 0.01) by treating the excitations as bosons^34,35^. In fact, the calculated spectrum resembles that observed in C4mimFeCl_4_, though the model used is based on an fcc lattice and any atomistic disorder is not considered. A recent theoretical paper also suggests localized soft plastic modes whose excitation intensity reaches zero in proportion to ω^4^ in a three-dimensional Heisenberg SG^36^. Therefore, we consider that the localized magnetic excitation is one of the inherent dynamics in SGs and can be observed in other spin glasses separated from magnetically ordered phases. Reinvestigation of SGs such as dilute magnetic alloys is required to clarify universality of the magnetic boson peak.

Finally, we briefly discuss the magnetic interaction within C4mimFeCl_4_. Magnetic structures of imidazolium-based MILs have been investigated by neutron diffraction^37–41^. Typically, they have two-dimensional structure constituting anion and cation layers. Here, the main magnetic couplings are via superexchange anion-anion interactions (Fe–Cl⋯Cl–Fe). As the sign of the interaction is determined by the superexchange angles, both AFM and FM interactions typically coexist. Actually, a related substance EdimimFeCl_4_ (1-ethyl-2,3-dimethylimidazolium tetrachloroferrate) has two intraplane interactions, nearest-neighbor FM and next-nearest-neighbor AFM interactions, and an interplane AFM interaction^40^. The structural analysis for C4mimFeCl_4_ has not yet been succeeded because the crystalline sample obtained by annealing has strong preferred orientations. Nevertheless, one can naturally consider that both AFM and FM interactions coexist in the structural glass of C4mimFeCl_4_ with atomic disorder. In this conjecture, spin clusters can be formed via the interactions below *T*_SG_, generating a multitude of localized magnetic modes whose energy is determined by the spin configuration of each spin cluster. Consequently, this system exhibits Bose-scaled broad magnetic excitations with a high energy tail.

## Methods

### Sample preparation and characterization

Hydrogenated C4mimFeCl_4_ (h-C4mimFeCl_4_) with purity specified as > 98% was purchased from Tokyo Chemical Industry and used for magnetization measurements and a neutron scattering experiment to investigate vibrational excitations. The deuterated analogue (d-C4mimFeCl_4_) was used to probe for magnetic scattering, to avoid strong incoherent scattering contribution from H atoms. d-C4mimFeCl_4_ was prepared via the procedure as follows. 1-Butyl-3-methylimidazolium chloride (C4mimCl) was first synthesized using a literature method with modifications^42^. Reagents: 1-methylimidazole-*d*_6_ (98% atom D, CDN isotopes), 1-chlorobutane-*d*_9_ (98% atom D, CDN isotopes), iron(III) chloride (98%, Alfa Aesar), super dehydrated toluene (99.5%, FUJIFILM Wako Pure Chemical Co., Ltd.), dichloromethane (99.5%, FUJIFILM Wako Pure Chemical Co., Ltd.), and deuterium oxide (99.9%-*d*, Sigma-Aldrich).

In an argon-filled glove bag, 1-methylimidazole-*d*_6_ (2.0 g, 22.7 mmol) was dissolved in dehydrated toluene (10 mL). 1-Chlorobutane-*d*_9_ (2.0 g, 19.7 mmol) was then added and the mixture was stirred at 20 °C with argon bubbling for 10 min. After, the resulting mixture was refluxed in an oil bath at 120 °C for 7 days. When the reaction was complete, the solvent was cooled to 20 °C and evaporated to dryness under reduced pressure. The crude product was washed a third time with dichloromethane (30 mL), and the remaining solution was evaporated to dryness. The yield of deuterated C4mimCl was 38.2% (1.53 g). The deuteration level was estimated to be greater than 99% by nuclear magnetic resonance and electrospray ionization mass spectrometry (Supplementary SI-1).

C4mimCl-*d*_15_ (0.97 g, 5.1 mmol) and FeCl_3_ (0.865 g, 5.3 mmol) were dissolved in 10 mL of D_2_O, and the mixture was stirred at 20 °C for 10 min. Then, the mixture was filtrated through a polypropylene filter (0.45 μm pore size) to remove the small amount of insoluble material. The filtered solution was evacuated at 70 °C for 24 h to dryness under reduced pressure. The yield of d-C4mimFeCl_4_ was 94.3% (1.73 g).

The thermodynamic and paramagnetic properties of both h- and d-C4mimFeCl_4_ were examined by differential scanning calorimetry (DSC) and magnetic susceptibility measurements. Deuterium isotope effects on these properties were not confirmed (Supplementary SI-2).

### Magnetization measurements

The magnetizations of C4mimFeCl_4_ were measured using a SQUID magnetometer (MPMS-XL, Quantum Design Co.) in the temperature range of 1.8–300 K. For the measurement sample with SQUID, liquid C4mimFeCl_4_ (0.1010 g) was put in a clear gelatin capsule. The capsule was fixed to a straw and set in the SQUID magnetometer. The dc magnetization measurements in the temperature ranges of 0.03–0.8 K and 0.4–3.0 K were performed using a capacitive Faraday magnetometer installed in a ^3^He and a dilution refrigerator, respectively^43^. In the Faraday method, a small cup made of pure copper whose surface is coated with gold was prepared. The liquid C4mimFeCl_4_ (0.0706 g) was poured into this cup and set using Apiezon N grease in the measurement stage. All the magnetization data were obtained under an applied magnetic field of 100 Oe.

### Neutron scattering measurements

Neutron scattering measurements were performed using a disk-chopper spectrometer AMATERAS^44^ and a near-backscattering TOF spectrometer DNA^45^ at the Materials and Life Science Experimental Facility (MLF), J-PARC. The d-C4mimFeCl_4_ sample was used to explore magnetic scattering and the h-C4mimFeCl_4_ sample for vibrational excitations. The deuterated sample was loaded into an annular Al can with a diameter of 7 mm, while the hydrogenated sample was placed into a concentric double-cylinder Al can with a diameter of 14 mm and a thickness of 0.5 mm. To control the temperature of the samples, a ^3^He refrigerator, top-loading closed-cycle ^4^He refrigerator, and dilution refrigerator were used. The glassy C4mimFeCl_4_ was created by cooling the liquid with a rate of 1–3 K/min. The crystalline sample was obtained by the following annealing procedure. The glassy sample was first kept at 130 K for 2 h and then heated to 230 K, which is 35 K below the melting temperature. The crystallization occurred at 230 K within 1 h, which was confirmed by the growth of the Bragg diffraction peaks.

In the measurements on AMATERAS for magnetic scattering, the incident neutron energies used were *E*_i_ = 3.13 and 1.68 meV with corresponding energy resolutions (∆*E*) of 62 and 26 µeV (full width at half maximum of the elastic peaks), respectively. The scattering signals were recorded in the temperature range of 0.3–100 K. The vibrational excitations were measured with *E*_i_ = 7.74 meV and ∆*E* = 0.22 meV at *T* = 7 K and 50 K. The DNA spectrometer having a higher energy resolution (∆*E* = 3.7 µeV) was used to investigate the magnetic excitation of glassy d-C4mimFeCl_4_ below ℏω = 0.27 meV in the temperature range of 0.3–0.5 K. The final neutron energy was fixed to 2.08 meV. Moreover, wavelength frame multiplication was utilized to extend the energy window while maintaining the energy resolution. All the data collected were analyzed using the software suite UTSUSEMI^46^. The dynamical structure factor *S*(*Q*,ω) shown in this article are obtained by subtracting elastic signals. The elastic scattering contribution is estimated by the convolution of resolution function and *I*(*Q*) of the sample. The resolution functions are obtained by the data of the crystal state at 0.3 K for AMATERAS and vanadium standard for DNA.

## Supplementary Information

Supplementary Information.
