# Supplementary material for: Spin glass behavior and magnetic boson peak in a structural glass of a magnetic ionic liquid
Source: Sci Rep. 2021 Jun 8;11:12098. doi: 10.1038/s41598-021-91619-z (PMC8187720; doi:10.1038/s41598-021-91619-z)
Supplement: Supplementary file 1 — Supplementary Information. [file 41598_2021_91619_MOESM1_ESM.pdf]

Supplementary Information for
Spin glass behavior and magnetic boson peak in a structural glass of a magnetic ionic liquid

Maiko Kofu,[1,*] Ryuta Watanuki,[2,†] Toshiro Sakakibara,[3] Seiko Ohira-Kawamura,[1] Kenji Nakajima,[1] Masato Matsuura,[4] Takeshi Ueki,[5] Kazuhiro Akutsu,[4] and Osamu Yamamuro[3]

[1] *J-PARC Center, Japan Atomic Energy Agency, Tokai, Ibaraki 319-1195, Japan*

[2] *Division of Materials Science and Chemical Engineering, Faculty of Engineering, Yokohama National University, Yokohama, Kanagawa 240-8501, Japan*

[3] *Institute for Solid State Physics, University of Tokyo, Kashiwa, Chiba 277-8581, Japan*

[4] *Comprehensive Research Organization for Science and Society, Tokai, Ibaraki 319-1106, Japan*

[5] *National Institute for Materials Science, Tsukuba, Ibaraki 305-0044, Japan*

SI-1. Estimate of deuteration level

To estimate the deuteration level of C4mim cation, NMR and ESI-MS measurements have been performed in the same way as in the previous report [S1]. NMR spectra were recorded using a JNM-ECA 500 spectrometer (JEOL Ltd., Japan). Figure S1 shows the ^{1}H-NMR (400 MHz) spectrum in D_2O with 1,4-dioxane (internal standard) and ^{2}H-NMR (77 MHz) in H_2O. The peaks from the sample can be clearly seen in ^{2}H-NMR, but are almost absent in ^{1}H-NMR. The peak positions are as follows: ^{1}H NMR (400 MHz, D_2O); δ 0.85 (residual signal), 1.16 (residual signal), 1.87 (residual signal), 3.87 (residual signal), 4.12 (residual signal), 7.59 (residual signal), 8.66 (residual signal); ^{2}H NMR (77 MHz, H_2O); δ 0.80 (brs), 1.18 (brs), 1.72 (brs), 3.85 (brs), 4.09 (brs), 7.39 (brs), 7.44 (brs), 8.66 (brs). The deuteration level of C4mim cation is estimated to be 99.6 % from the ^{1}H-NMR data.

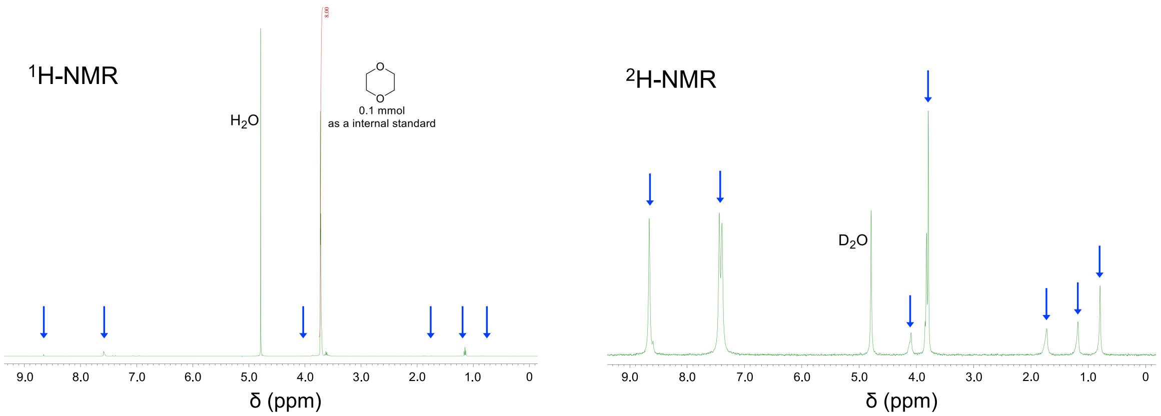

Figure S1. ^{1}H-NMR (400 MHz, D_2O) and ^{2}H-NMR (77 MHz, H_2O) spectra of deuterated C4mimCl. Arrows indicate the positions of ^{1}H or ^{2}H chemical peak.

Figure S2 presents an ESI-MS spectrum, showing the mass distribution of the different isotopologues (d_{12} - d_{15}), recorded on an EXTREMA-MS-100P (Nihon Bunko Co. Ltd., Japan) spectrometer. The distribution of the isotopologues is as follows (M^+): 5.0 %, d_{12}; 19.8 %, d_{13}; 50.0 %, d_{14}; 25.1 %, d_{15}. Considering the fact that the deuterium atom at the C1 position is easily exchanged with a proton of the ESI-MS solvent (H_2O-EtOH mixture), the deuteration level of the C4mim cation is estimated to be over 99 %, which is consistent with the NMR result.

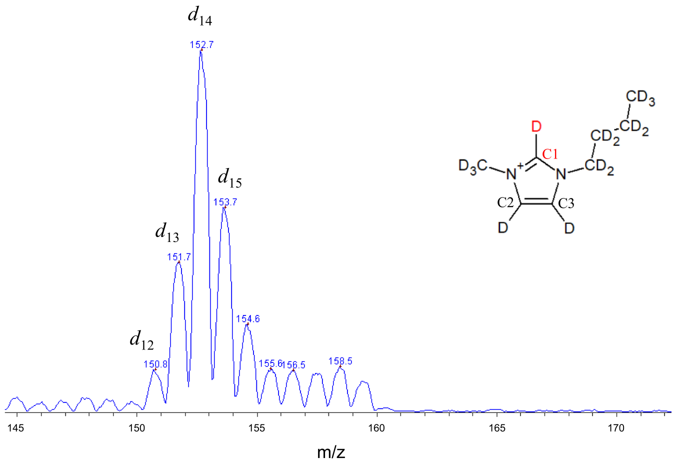

Figure S2. Electrospray ionization mass spectra in positive mode of C4mim cation.

SI-2. Thermodynamic and paramagnetic properties of deuterated sample

DSC curves obtained using DSC7020 (Seiko Instruments Inc., Japan) and magnetic susceptibilities by MPMS (Quantum Design, USA) are shown in Fig. S3. For both the hydrogenated and deuterated samples, the glass transition occurred at $\sim$ 190 K and an anomaly due to partial crystallization was detected at around 270 K. These results are consistent with those obtained by adiabatic calorimetry [10]. In addition, no significant difference in the paramagnetic behavior was found between the two samples. Therefore, deuterium isotope effects on these properties were not confirmed.

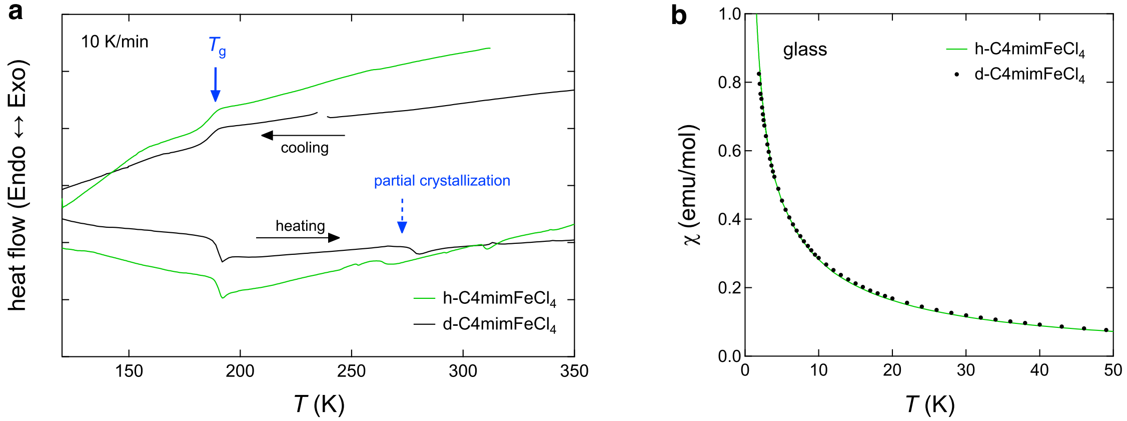

Figure S3. **a**, DSC curves and **b**, magnetic susceptibilities of the structural glass state for hydrogenated and deuterated C4mimFeCl$_4$.

SI-3. Curie-Weiss fit

Figure S4 shows the inverse molar susceptibilities χ^{-1} of glassy and crystalline C4mimFeCl$_4$. The χ^{-1} data in the paramagnetic region were fitted with a Curie-Weiss law $\chi^{-1} = (T - \theta)/C$, where C is the Curie constant $(= N_A \mu^2 \mu_B^2 / 3k_B)$. The obtained values of the Weiss temperatures (θ) and effective magnetic moments (μ) are $\theta = -3.58(5)$ K and $\mu = 5.567(1)$ μ_B for the glass state and $\theta = -4.08(16)$ K and $\mu = 5.564(2)$ μ_B for the crystal state, respectively. Both the glassy and crystalline samples have nearly the same values of θ and μ, indicating a similar magnetic interaction energy.

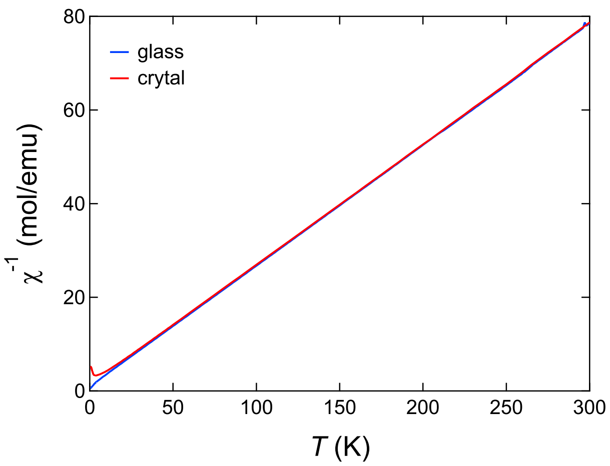

Figure S4. Inverse molar magnetic susceptibilities of C4mimFeCl_4 obtained at $H = 100$ Oe in both the structural glass and crystal states.

SI-4. Time-dependence of magnetization

The time evolution of the magnetization in the aging regime was investigated using a protocol introduced by the previous literature [13]. Figure S5 presents the ZFC magnetizations against time with intermittent stops. The magnetization with one stop at 0.2 K gradually grows with time and saturates after 1,000 s (Fig. S5a). The time evolution can be well fitted by a stretched exponential function, $M = M_0\{1 - \exp[-(t/\tau)^\beta]\}$, with $\beta = 0.33 \pm 0.02$ (blue curve). Attempts to fit the data with $\beta = 1$ was not successful (green curve), indicating non-Debye relaxation. The small value of β suggests a broad distribution of relaxation times due to the presence of a number of degenerate metastable states, which is a characteristic feature of SGs. Note that superparamagnetic materials with two ground states exhibit Debye-type relaxation.

The blue solid circles in Fig. S5b represent the magnetization with two stops at 0.18 K by 580 s and at 0.2 K. In the two-stop process, the magnetization increases and saturates to a certain value below the saturation value with one stop at 0.2 K. After the sample is heated to 0.2 K, the magnetization grows again. When the one-stop curve is shifted to the right by 380 s (magenta open circles), it almost coincides with the data of the two-stop curve at 0.2 K. Therefore, the state annealed at a certain temperature does not depend on the thermal history.

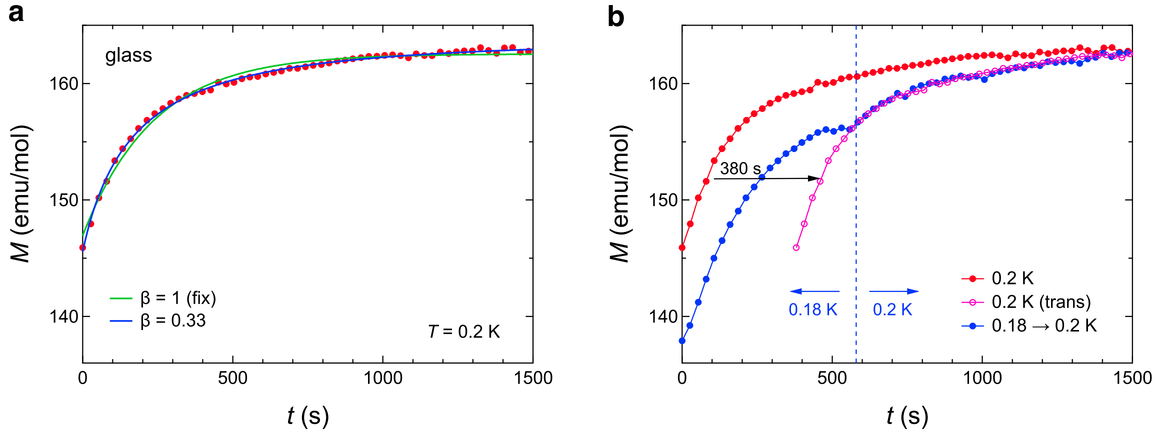

Figure S5. **a**, ZFC magnetizations against time with one intermittent stop. Solid curves represent stretched exponential fits to the data obtained by $\beta = 1$ (green) and by leaving β free (blue). **b**, ZFC magnetizations against time with one (red solid circles) and two (blue solid circles) stops. Magenta open circles denote the one-stope data shifted along the horizontal axis by 380 s. All the data were collected for $H = 100$ Oe.

SI-5. Comparison of neutron scattering data between hydrogenated and deuterated samples

Figure S6a shows the diffraction patterns of hydrogenated (h) and deuterated (d) $C4mimFeCl_4$. As H atoms are replaced with D atoms, the Q-independent signals, i.e., incoherent scattering, are significantly reduced. The magnetic Bragg peaks (red arrows) were detected in both isotopes.

The energy spectra of the samples are summarized in Figs. S6b,c. A clear difference is observed between the isotopes in the crystal state. Sharp excitations appear at ±0.08 meV in crystalline h-$C4mimFeCl_4$, which is attributed to rotational tunneling of the methyl group [S2]. As the probability of tunneling is reduced upon deuteration, the excitations are absent in d-$C4mimFeCl_4$. Meanwhile, in the spin glass state, the spectra are similar between the isotopes, except in the range below 0.1 meV. The invisibility of the tunneling excitations is owing to the three-fold symmetry breaking. However, we cannot exclude the possibility of the presence of tunneling excitation with the distribution of excitation energy. The higher intensity below 0.1 meV in h-$C4mimFeCl_4$ could be due to a tail of strong elastic scattering and/or the tunneling excitation.

Although the magnetic scattering is visible in h-$C4mimFeCl_4$, the use of the deuterated analogue is necessary to unambiguously examine the spin dynamics, particularly in the glass state because the separation of magnetic scattering from nuclear scattering is not straightforward.

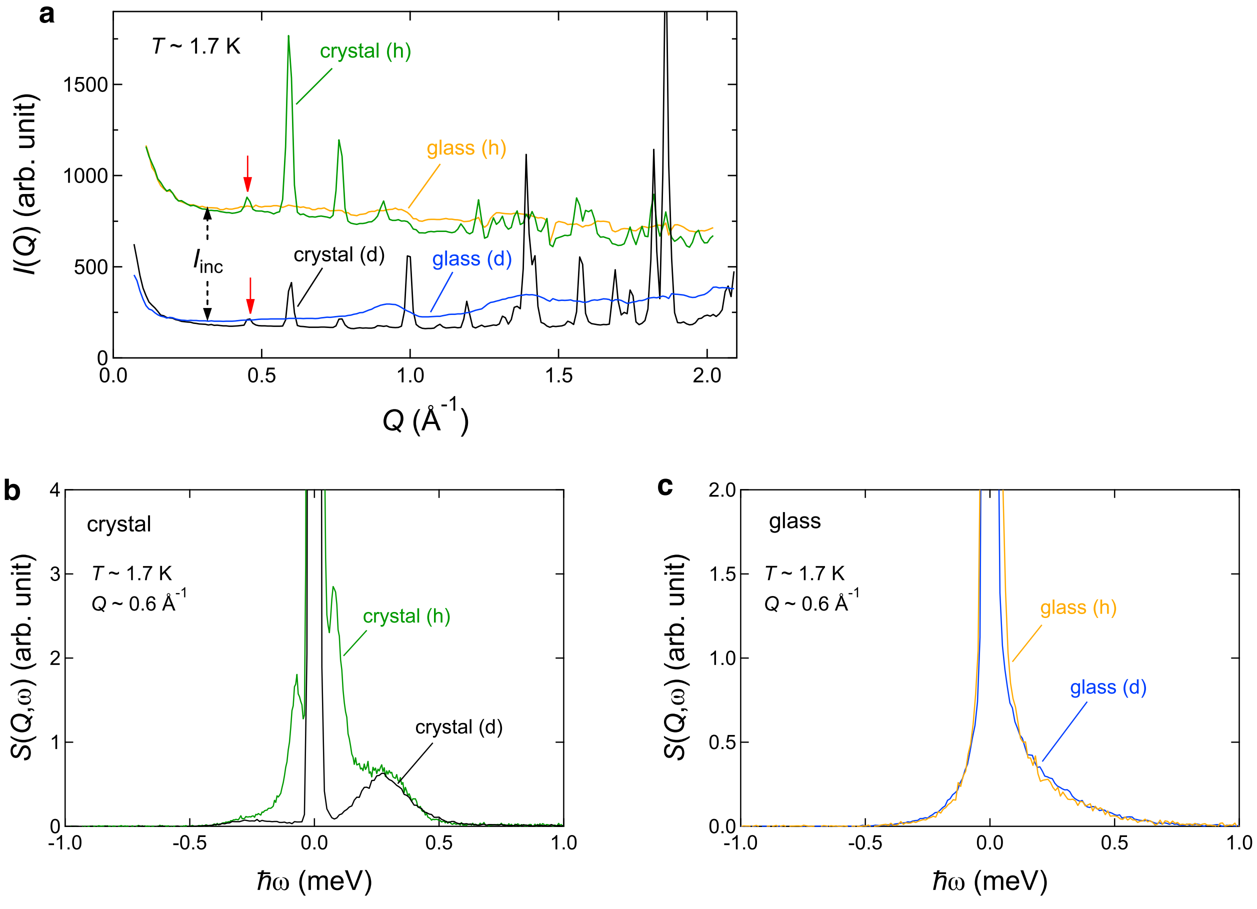

Figure S6. **a**, Diffraction patterns of h- and d-$C4mimFeCl_4$ for the glassy and crystal states at $T \sim 1.7$ K. Red arrows indicate the positions of magnetic Bragg peaks; black dashed arrows represent the incoherent scattering, mainly from H atoms (I_{inc}). **b**, **c**, Energy dependence of dynamical structure factors at $Q \sim 0.6$ Å^{-1} and $T \sim 1.7$ K for (**b**) crystal and (**c**) structural glass.

SI-6. Elastic magnetic signals

Figure S7 displays a difference in the elastic intensity $I(Q)_{0.3\mathrm{K}} - I(Q)_{10\mathrm{K}}$, where only magnetic contributions are shown. The magnetic diffuse peak in the structural glass appears at $Q \sim 0.6$ Å^{-1}, at which magnetic Bragg peaks appear in the crystalline sample. This indicates that the local spin configuration of the SG state is similar to that of the AFM phase. The distance between Fe ions in the structural glass may be almost the same as that in the crystal.

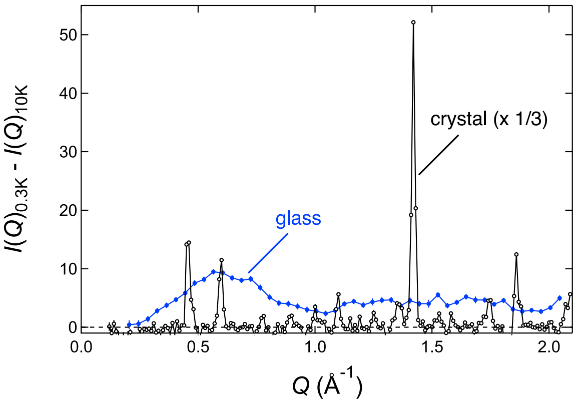

Figure S7. Difference in elastic intensity between 0.3 K and 10 K for the glass and crystal states. The data of the crystal state are multiplied by 1/3 for the sake of clarity.

SI-7. Temperature evolution of $S(Q,\omega)$ maps

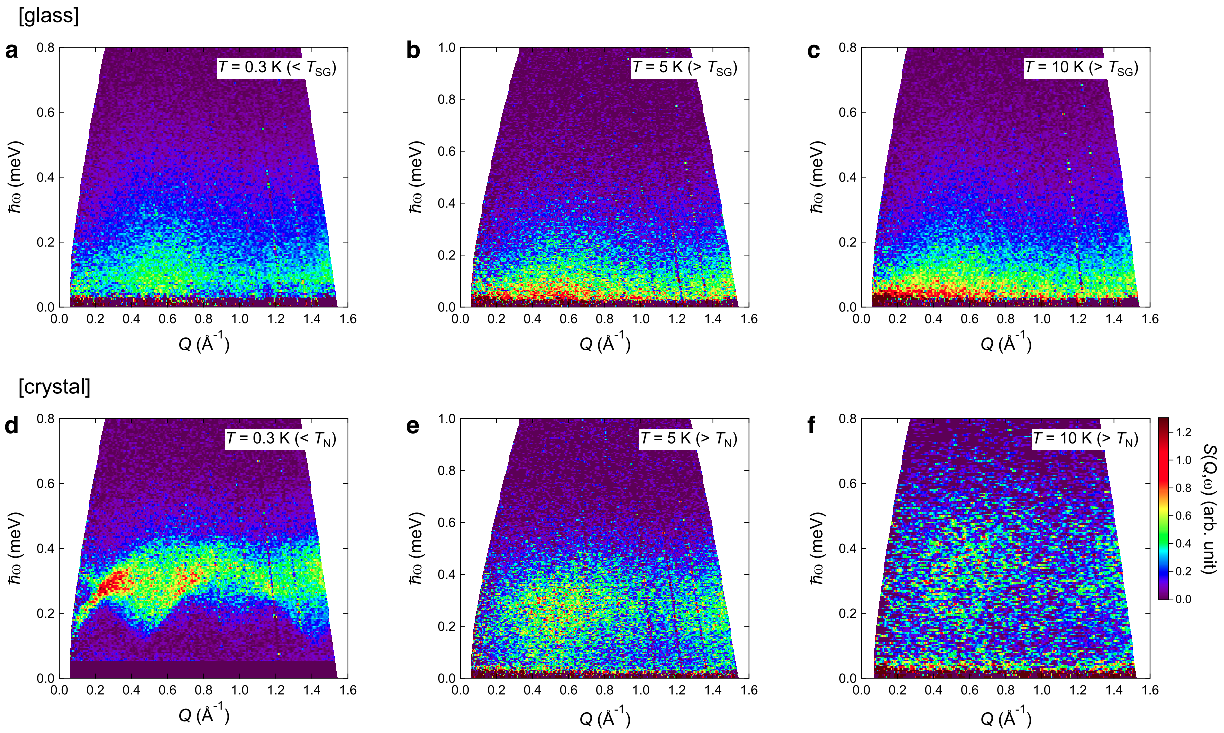

Figure S8. **a**, **b**, **c**, $S(Q,\omega)$ maps of glass state at 0.3 K (**a**), 5 K (**b**) and 10 K (**c**). **d**, **e**, **f**, $S(Q,\omega)$ maps of crystal state at 0.3 K (**d**), 5 K (**e**) and 10 K (**f**).

$S(Q,\omega)$ maps at 0.3, 5, and 10 K for both glass and crystal states are shown in Fig. S8. In glassy $C4mimFeCl_4$, the low-energy excitation is observed at 0.3 K ($< T_{SG}$). Above 5 K ($\approx 12T_{SG}$), the magnetic scattering becomes strong near $\hbar\omega = 0$, which is suggestive of the emergence of magnetic relaxation process (quasielastic scattering). More specifically, the system exhibits a liquid-like behavior above T_{SG}. In the crystalline sample, the excitation spectrum drastically changes in the temperature range. At 5 K ($\approx 2T_N$), a broad magnetic excitation is still visible. The magnetic scattering is spread over a wide $Q - \hbar\omega$ region at 10 K ($\approx 4T_N$). Apparently, the spin dynamics at 10 K is different between the glass and crystal states. This is related to the difference in magnetic susceptibility at 10 K, which is shown in Fig. 1b in the main text. We expect that the feature of $S(Q,\omega)$ is almost identical in both states above 50 K.

SI-8. Energy spectra

Figure S9 presents the temperature evolution of energy spectrum of glassy $C4mimFeCl_4$. The spectra shown here were obtained by subtracting an elastic contribution estimated from the data of the crystal state at 0.3 K, exhibiting the clear energy gap (see Fig. 3b in the main text and Fig. S10). The spectrum changes with temperature over the whole temperature range ($0.3 \leq T \leq 10$ K). It should be emphasized that it changes even below T_{SG}, mainly at low

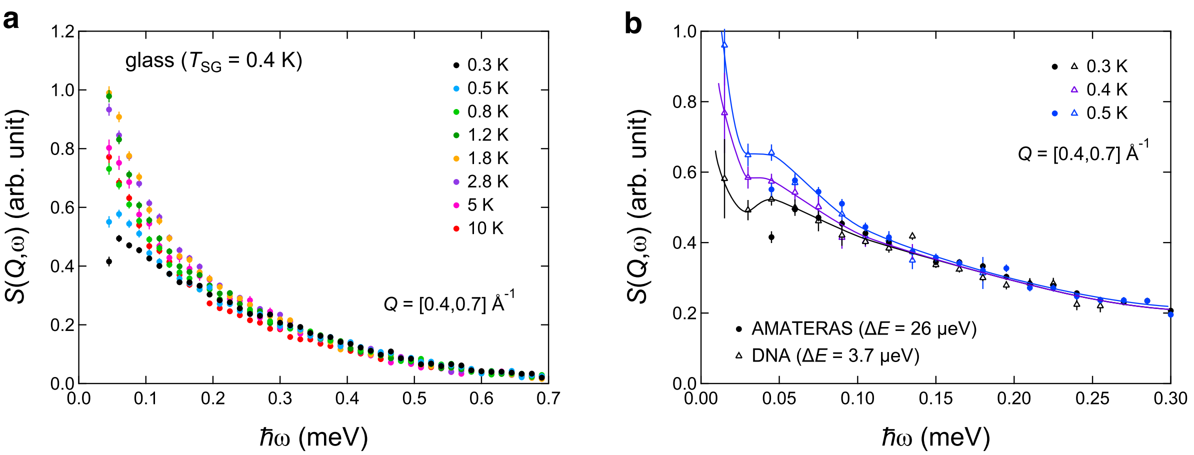

Figure S9. **a**, **b**, Energy spectra of $S(Q,\omega)$ of glassy $C4mimFeCl_4$ at $Q = [0.4, 0.7]$ Å^{-1} in (**a**) a wide temperature range and (**b**) below $T \approx T_{SG}$. Solid curves in **b** are a guide for the eye.

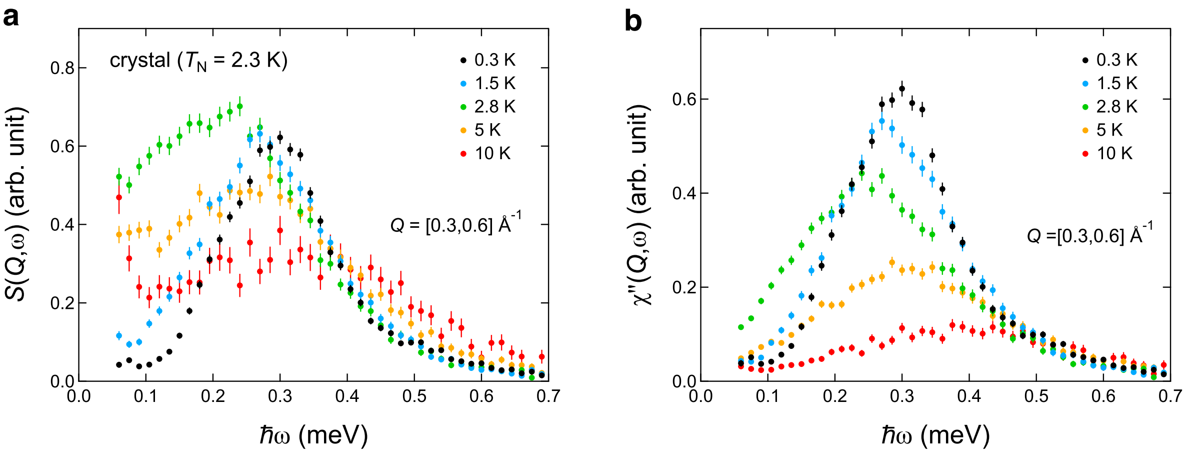

Figure S10. **a**, **b**, Energy spectra of (**a**) $S(Q,\omega)$ and (**b**) $\chi''(Q,\omega)$ of crystalline $C4mimFeCl_4$.

energies. As the Bose factor-corrected spectra remain the same below T_{SG} (Fig. 4a,b in the main text), the difference is ascribed to the Bose factor. Meanwhile, a different feature is observed in the crystalline sample (Fig. S10). At 0.3 K, the magnetic excitations with the energy gap of $\sim$ 0.1 meV are clearly seen at 0.3 K. The energy gap is reduced when approaching T_N and the paramagnetic scattering starts to appear above T_N. However, there seems to exist a broad excitation even at 10 K.

We mention here that the excitations at 0.3 K exist up to $\sim$ 0.5 meV for both the glassy and crystal states. As the highest excitation energy reflects nearest-neighbor interactions, it implies that the interaction between the nearest-neighbor Fe^{3+} spins is nearly the same between the states. This is consistent with the fact that the Weiss temperature and effective moment are almost identical in the two states (see SI-3).

SI-9. Contribution from nuclear scattering

To assess the contribution of nuclear scattering in the inelastic channel, i.e., vibrational excitations in the case well below T_g, the energy spectra at $Q = 0.65$ and 1.65 $Å^{-1}$ are plotted in Fig. S11. The scattering signal below 1 meV is higher at lower Q and decreases upon heating, which is attributed to magnetic scattering. While the temperature-independent scattering is observed above 1 meV and is stronger at higher Q. This scattering is vibrational excitations, that is, the boson peak described in SI-11. Therefore, the inelastic signals of d-C4mimFeCl$_4$ at $\hbar\omega \leq 1$ meV, $Q \leq 1$ $Å^{-1}$ and $T \leq 10$ K, which we focus on in this article, mostly arise from magnetic scattering.

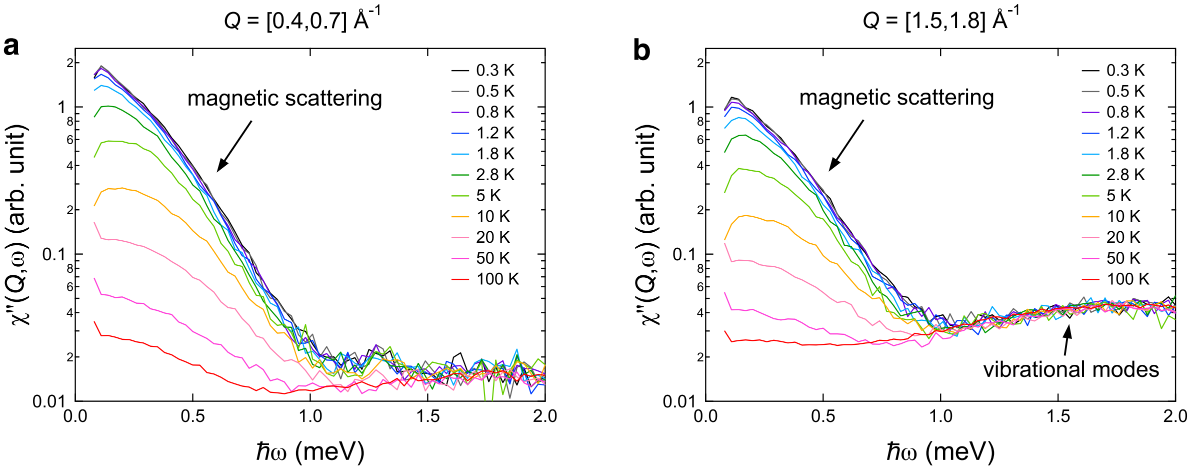

Figure S11. **a**, **b**, Energy spectra of (**a**) $\chi''(Q,\omega)$ at $Q = [0.4, 0.7]$ $Å^{-1}$ and (**b**) $[1.5, 1.8]$ $Å^{-1}$ in glassy C4mimFeCl$_4$.

SI-10. Structural boson peak

The structural boson peak in the glassy C4mimFeCl$_4$ was also investigated, where the hydrogenated sample was used to easily detect vibrational excitations. Figure S12 shows the energy spectra of $\chi''(Q,\omega)$ taken at 7 K and 50 K. It is apparent that the spectra exhibit a peak at $\sim$ 2.6 meV, which is a typical value of boson peak energy in ionic liquids [S3]. The spectrum remains almost the same at the two temperatures and thus are Bose-scaled. The slight difference below 3 meV between 7 K and 50 K is due to the occurrence of a fast relaxation process. It is known that there exist multiple relaxation processes in ionic liquids, reflecting their hierarchical structure and dynamics [S4].

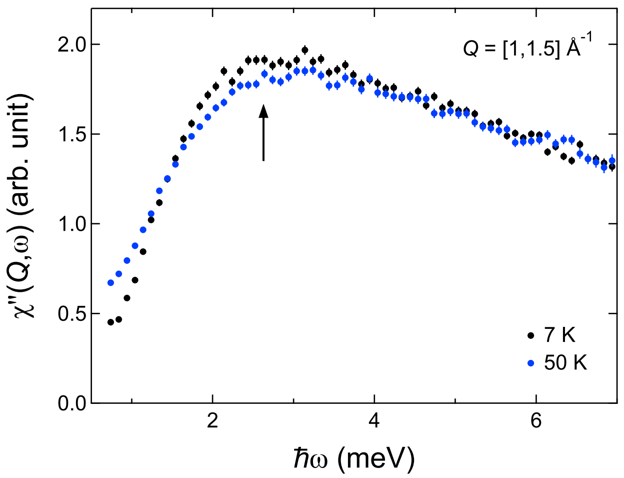

Figure S12. Energy spectra of $\chi''(Q, \omega)$ at $Q = [1, 1.5]$ Å^{-1} in glassy C4mimFeCl$_4$ at $T = 7$ and 50 K. Arrow indicates the position of the boson peak energy.

---

* Corresponding email: maiko.kofu@j-parc.jp
† Corresponding email: watanuki-ryuta-sm@ynu.ac.jp

[S1] Akutsu-Suyama, K. et al. Controlled deuterium labelling of imidazolium ionic liquids to probe the fine structure of the electrical double layer using neutron reflectometry. *Phys. Chem. Chem. Phys.* **21**, 17512 (2019).

[S2] Prager, M. & Heidemann, A. Rotational Tunneling and Neutron Spectroscopy: A Compilation. *Chem. Rev.* **97**, 2933-2966 (1997).

[S3] Kofu, M. et al. Inelastic neutron scattering study on boson peaks of imidazolium-based ionic liquids. *J. Mol. Liq.* **210**, 164-168 (2015).

[S4] Kofu, M. et al. Quasielastic neutron scattering studies on glass-forming ionic liquids with imidazolium cations. *J. Chem. Phys.* **143**, 234502 (2015).
